# Rapid clinical validation of an RNA/DNA hybrid tagmentation-based metagenomic workflow for respiratory RNA virus detection

**DOI:** 10.3389/fmicb.2026.1849991

**Published:** 2026-07-15

**Authors:** Xia Ma, Shan Guo, Yangyang Feng, Murong Su, Feili Wei, Xuejun Liu

**Affiliations:** 1 First Hospital of Shanxi Medical University, Taiyuan, Shanxi, China; 2 Beijing Shijingshan Hospital, Beijing, China; 3 Beijing Institute of Hepatology, Beijing YouAn Hospital, Capital Medical University, Beijing, China; 4 Department of Public Health Sciences, University of California, Irvine, Irvine, CA, United States

**Keywords:** clinical validation, metagenomic next-generation sequencing, respiratory RNA viruses, RNA/DNA hybrid tagmentation, syndromic surveillance

## Abstract

**Background:**

In the post-pandemic era, co-circulation of multiple respiratory RNA viruses has increased the need for timely diagnosis and reliable recognition of mixed infections. Although reverse transcription quantitative polymerase chain reaction (RT-qPCR) remains the clinical standard for respiratory virus detection, its target-restricted design limits the detection of unexpected or coinfecting pathogens. Conventional metagenomic next-generation sequencing (mNGS) provides hypothesis-free pathogen detection, but routine clinical use is still limited by long turnaround times and complex library preparation. Therefore, a sequencing-based strategy that preserves broad, unbiased detection while offering a simplified workflow and clinically acceptable turnaround time is needed.

**Methods:**

We optimized and clinically validated CATCH, a rapid RNA/DNA hybrid tagmentation-based mNGS workflow, for respiratory RNA virus detection. Analytical performance was assessed using standardized reference materials, including SARS-CoV-2 and influenza A virus, with evaluations of sensitivity, reproducibility, short-term stability, and host-background interference. Clinical validation was performed in retrospective and prospective respiratory infection cohorts, and assay performance was benchmarked against RT-qPCR and multiplex PCR. The same sequencing data were further examined for semiquantitative viral assessment, coinfection detection, and exploratory respiratory microbial profiling.

**Results:**

The optimized CATCH workflow shortened library preparation to approximately 3 h, with about 35 min of hands-on time, enabling same-day sequencing-based diagnostics. Broad detection was achieved across seven clinically relevant respiratory RNA viruses. Sequencing-derived viral abundance showed a significant overall correlation with viral input concentration, supporting semiquantitative interpretation, although virus- and subtype-specific variability highlighted biological constraints on absolute quantification. Using SARS-CoV-2 and influenza A virus as representative targets, CATCH achieved clinically actionable limits of detection with high reproducibility and stability. In clinical cohorts, CATCH showed high concordance with routine molecular assays and identified mixed respiratory infections missed by targeted testing. Exploratory analyses also demonstrated the feasibility of respiratory microbial community profiling from the same sequencing dataset.

**Conclusion:**

CATCH is a rapid and clinically deployable RNA virus mNGS workflow that helps bridge targeted molecular diagnostics and conventional metagenomic sequencing. By combining broad pathogen detection, coinfection identification, and semiquantitative assessment within a streamlined workflow, CATCH provides a practical framework for comprehensive respiratory RNA virus diagnosis and syndromic surveillance.

## Introduction

Respiratory tract infections remain a leading cause of global morbidity and mortality, particularly among vulnerable populations, including older adults, children, and immunocompromised individuals. The COVID-19 pandemic has reshaped respiratory virus surveillance and clinical diagnostics by accelerating the adoption of molecular testing while also exposing its limitations. In the post-pandemic era, co-circulation of multiple respiratory RNA viruses, including SARS-CoV-2, influenza A and B viruses, respiratory syncytial virus (RSV), human metapneumovirus (hMPV), and parainfluenza viruses, has become increasingly common. This changing epidemiological landscape poses new challenges for timely diagnosis, infection control, and clinical decision-making (World Health Organization, 2024; World Health Organization, 2025; Carter et al., 2022).

Reverse transcription quantitative polymerase chain reaction (RT-qPCR) remains the clinical standard for respiratory virus detection because of its high sensitivity and rapid turnaround time. However, PCR-based assays are inherently target-restricted and require continual updating as viral diversity evolves. Multiplex PCR panels partially expand diagnostic coverage, but they remain constrained by predefined target lists and have limited capacity to detect unexpected, rare, or emerging viruses, as well as coinfections involving multiple pathogens. These limitations are particularly relevant in severe pneumonia, immunocompromised hosts, and intensive care unit (ICU) patients, in whom atypical pathogens and mixed infections are more frequently encountered (Gaston, 2023; Tan et al., 2024; Gauthier et al., 2024).

Metagenomic next-generation sequencing (mNGS) offers a hypothesis-free approach for detecting a broad spectrum of pathogens in a single assay and can also generate genomic information for strain typing and mutation analysis (Chiu and Miller, 2019; Gaston, 2023; Chen et al., 2024; Jin et al., 2022; Yang et al., 2022; Zheng et al., 2024). Despite these advantages, conventional mNGS workflows remain difficult to implement routinely because of prolonged turnaround times, labor-intensive library preparation, high cost, and substantial host-background interference. RNA virus detection is especially challenging because standard workflows usually require multistep reverse transcription, second-strand synthesis, and library construction procedures, which limit scalability in time-sensitive clinical settings (Tan et al., 2024; Gauthier et al., 2024; Zhu et al., 2025).

To address these limitations, we previously developed an RNA/DNA hybrid tagmentation-based mNGS strategy using Tn5 transposase, termed CATCH, which enables direct library construction from RNA/DNA hybrids and substantially simplifies RNA virus sequencing (Di et al., 2020; Lu et al., 2020; Zhang et al., 2021; Wei et al., 2020). Those earlier studies established the technical feasibility of RNA/DNA hybrid tagmentation for RNA virus detection. However, whether this approach could meet the practical requirements of clinical respiratory virus diagnosis had not been systematically evaluated, particularly with regard to shortened library preparation, reproducible analytical sensitivity, host-background tolerance, semiquantitative interpretation, and validation in real-world clinical cohorts.

In this study, we further optimized and clinically validated the CATCH workflow as a rapid, deployable mNGS approach for respiratory RNA virus detection, with reference to established principles for clinical metagenomic assay validation (Schlaberg et al., 2017). Compared with the previously reported feasibility-oriented workflow, the optimized protocol directly uses first-strand RNA/DNA hybrids for Tn5-based tagmentation, avoids a separate second-strand synthesis step, and reduces library preparation time to approximately 3 h with minimal hands-on input. We systematically evaluated its analytical performance using standardized reference materials and clinical respiratory specimens, including limit of detection, reproducibility, short-term stability, host-background interference, and semiquantitative performance. We also examined its capacity for subtype discrimination and mixed-infection detection across multiple clinically relevant respiratory RNA viruses.

Finally, using retrospective and prospective clinical cohorts, we benchmarked the diagnostic performance of CATCH against RT-qPCR and multiplex PCR assays and explored whether additional respiratory microbial information could be obtained from the same sequencing data. Together, these analyses position CATCH as a practical bridge between targeted molecular diagnostics and conventional mNGS, defining its clinical performance boundaries for rapid respiratory virus diagnosis and syndromic surveillance (Tan et al., 2024; Gauthier et al., 2024).

## Methods

### Study design and workflow overview

The optimized CATCH workflow was evaluated through analytical validation using standardized reference materials and clinical validation in retrospective and prospective respiratory infection cohorts, following commonly reported validation elements for clinical metagenomic workflows (Schlaberg et al., 2017). All samples were processed using a unified laboratory workflow and bioinformatics pipeline to ensure consistency and comparability across experiments.

To clarify the relationship between the present workflow and the previously reported CATCH protocol, we compared the two workflows in terms of workflow structure, library preparation time, hands-on time, validation design, and clinical evaluation scope. Because the earlier and current studies used different sample sets, reference materials, and validation frameworks, a formal head-to-head comparison of diagnostic sensitivity and specificity was not performed. The present study was therefore designed to define the analytical and clinical performance of the optimized workflow under the current validation framework. The main differences between the previous and optimized workflows are summarized in Supplementary Table S1.

### Ethics statement

This study was approved by the Ethics Committee of Beijing YouAn Hospital, Capital Medical University ([LL-2021-162-K]). Written informed consent was obtained from prospective participants or their legal guardians, as appropriate. For the retrospective analysis of residual clinical specimens, the requirement for informed consent was waived in accordance with institutional and national regulations.

### Sample preparation and RNA extraction

Respiratory samples were oropharyngeal swabs collected from patients with suspected viral respiratory infection and from healthy volunteers at Beijing YouAn Hospital, Capital Medical University as part of routine diagnostic care. Viral RNA was extracted using the QIAamp Viral RNA Mini Kit (Qiagen, Cat. No. 52904) according to the manufacturer’s instructions.

To monitor extraction efficiency and downstream assay performance, an internal RNA control, *Escherichia coli* bacteriophage MS2 (ATCC 15597-B1; Hecin Scientific), was added to each sample before extraction at a final concentration of 1 × 10^3^ copies/mL, corresponding to approximately tenfold the assay limit of detection (Tan et al., 2024; Gauthier et al., 2024).

Residual genomic DNA was removed using the denaturation buffer and DNase supplied in the REPLI-g WTA Kit (Qiagen, Cat. No. 150063). Following DNA depletion, RNA/DNA hybrids were generated using the SuperScript IV First-Strand Synthesis System (Thermo Fisher Scientific, Cat. No. 18091050) according to the manufacturer’s instructions (Di et al., 2020; Lu et al., 2020).

### Library construction using RNA/DNA hybrid tagmentation

RNA/DNA hybrid tagmentation and indexed library amplification were performed using an optimized protocol adapted from the Nextera XT DNA Library Prep Kit (Illumina, Cat. No. FC-131-1096). Compared with the previously described CATCH workflow, optimization focused on consolidating enzymatic steps, reducing amplification and handling time, and preserving library complexity and viral detection sensitivity.

After DNase treatment and first-strand synthesis, the resulting RNA/DNA hybrids were directly subjected to Tn5-based tagmentation without an additional second-strand synthesis step. Indexed library amplification was then performed after tagmentation, followed by magnetic bead purification and library quality assessment. The main procedural change was the consolidation of RNA/DNA hybrid formation, direct hybrid tagmentation, and indexed amplification into a shortened workflow. This reduced the total library construction time to approximately 3 h, with approximately 35 min of hands-on time. Detailed reagent information, incubation conditions, and step durations are provided in the Supplementary material (Di et al., 2020; Lu et al., 2020; Zhang et al., 2021).

Individual libraries were quantified using the Qubit dsDNA High Sensitivity Assay Kit (Thermo Fisher Scientific, Cat. No. Q32851) and pooled at equimolar concentrations. Fragment size distribution of the pooled libraries was assessed using the High-Sensitivity DNA Kit on an Agilent 4,200 TapeStation (Agilent Technologies). Sequencing was performed on the Illumina NextSeq CN500 platform using single-end 75-cycle sequencing, generating approximately 10–20 million reads per library.

### Bioinformatics processing and pathogen identification

Raw sequencing reads were processed using a standardized bioinformatics pipeline. Briefly, low-quality reads and adapter sequences were removed using fastp (Chen et al., 2018). Low-complexity reads were filtered using Komplexity (Clarke et al., 2019). Host-derived reads were removed using BMTagger, and ribosomal RNA reads were excluded using SortMeRNA (Kopylova et al., 2012).

Taxonomic classification was performed using Kraken2 (v2.0.8-beta; confidence threshold = 0.2) against a comprehensive nucleotide reference database supplemented with the SARS-CoV-2 reference genome (NC_045512.2) (Wood et al., 2019). For non-SARS-CoV-2 viruses, reads assigned to each viral species were aligned to reference genomes using tblastx from the BLAST+ suite (Camacho et al., 2009). Alignments with ≥90% sequence identity and ≥90% query coverage were retained. Duplicate reads with identical genomic start positions were removed before abundance estimation.

For SARS-CoV-2, reads classified as Betacoronavirus were aligned against a curated Betacoronavirus reference set using blastn from the BLAST+ suite (Camacho et al., 2009), and best-hit reads mapping to SARS-CoV-2 were retained. Viral abundance was normalized as duplicate-filtered reads per million total reads (RPM).

Influenza A virus subtyping was performed using the TAEC pipeline with the NCBI influenza virus database (Bao et al., 2008). A pathogen was considered positive when at least three nonoverlapping reads from distinct genomic regions were detected and the corresponding viral RPM was at least tenfold higher than that in no-template controls. Taxa not meeting these criteria were considered background contaminants and excluded from downstream analyses (Fierer et al., 2025; Salter et al., 2014).

#### Reference materials and analytical validation design

Analytical performance was evaluated using standardized reference materials, including the SARS-CoV-2 RNA Reference Standard (Lot No. GBW(E)091090; National Institute of Metrology, China) and the Second National Reference Panel for Influenza A Viral Nucleic Acid Detection (Lot No. 370051-201801; National Institutes for Food and Drug Control, China).

Seven respiratory RNA viruses and three influenza A virus subtypes were included in the analytical evaluation. For viruses without absolute concentration values, baseline input levels were defined as the highest dilution at which consistent detection was achieved. Viral inputs were then increased tenfold relative to baseline to assess semiquantitative performance (Tan et al., 2024; Zhu et al., 2025). All experiments were performed in triplicate.

### Analytical performance evaluation

The limits of detection (LODs) for SARS-CoV-2 and influenza A virus were determined using respiratory sample matrices spiked with serial dilutions of reference standards. The SARS-CoV-2 concentration ranged from 10 to 10^4^ copies per test, and the influenza A virus concentration ranged from 10^2^ to 10^5^ copies/mL. Probit regression analysis was performed in R to estimate the 95% LOD in accordance with CLSI EP17 guidelines (CLSI, 2012).

Assay precision was evaluated by within-run and between-run reproducibility testing using external positive controls and no-template controls across multiple sequencing runs. Short-term stability was assessed by storing extracted RNA samples at 4 °C for 0, 3, and 6 days before library construction. To evaluate host-background interference, SARS-CoV-2 and influenza A virus reference materials were spiked into samples containing low (1 × 10^4^), medium (1 × 10^5^), or high (1 × 10^6^) concentrations of A549 human cells (Gao et al., 2025; Fierer et al., 2025).

### Clinical samples and validation cohorts

Clinical validation was performed using both retrospective and prospective respiratory infection cohorts. The retrospective cohort included residual RNA samples collected in 2019 from patients with suspected COVID-19 at Beijing YouAn Hospital, Capital Medical University, with diagnostic results determined by RT-qPCR. A total of 98 samples from 64 patients were analyzed. Oropharyngeal swabs from 15 healthy volunteers were included as controls.

The prospective cohort consisted of 63 respiratory samples collected between November 2023 and February 2024 from patients with suspected respiratory infection. Samples were tested in parallel using CATCH and routine multiplex PCR assays. Mixed infections identified by CATCH were further evaluated using targeted multiplex PCR assays for confirmation (Tan et al., 2024; Gauthier et al., 2024).

### Statistical analysis

Statistical analyses were performed in R (v4.2.2). Continuous variables were compared using the Wilcoxon rank-sum test. Correlations between sequencing-derived abundance and RT-qPCR cycle threshold (Ct) values were assessed using Spearman’s rank correlation analysis when paired quantitative data were available. Diagnostic agreement between CATCH and routine molecular assays was assessed using 2 × 2 contingency tables. Sensitivity, specificity, and overall concordance were calculated using RT-qPCR or multiplex PCR as the reference standard, as appropriate. A two-sided *p* value < 0.05 was considered statistically significant.

## Results

### Optimized CATCH enabled rapid, same-day detection of diverse respiratory RNA viruses

**Figure 1:**
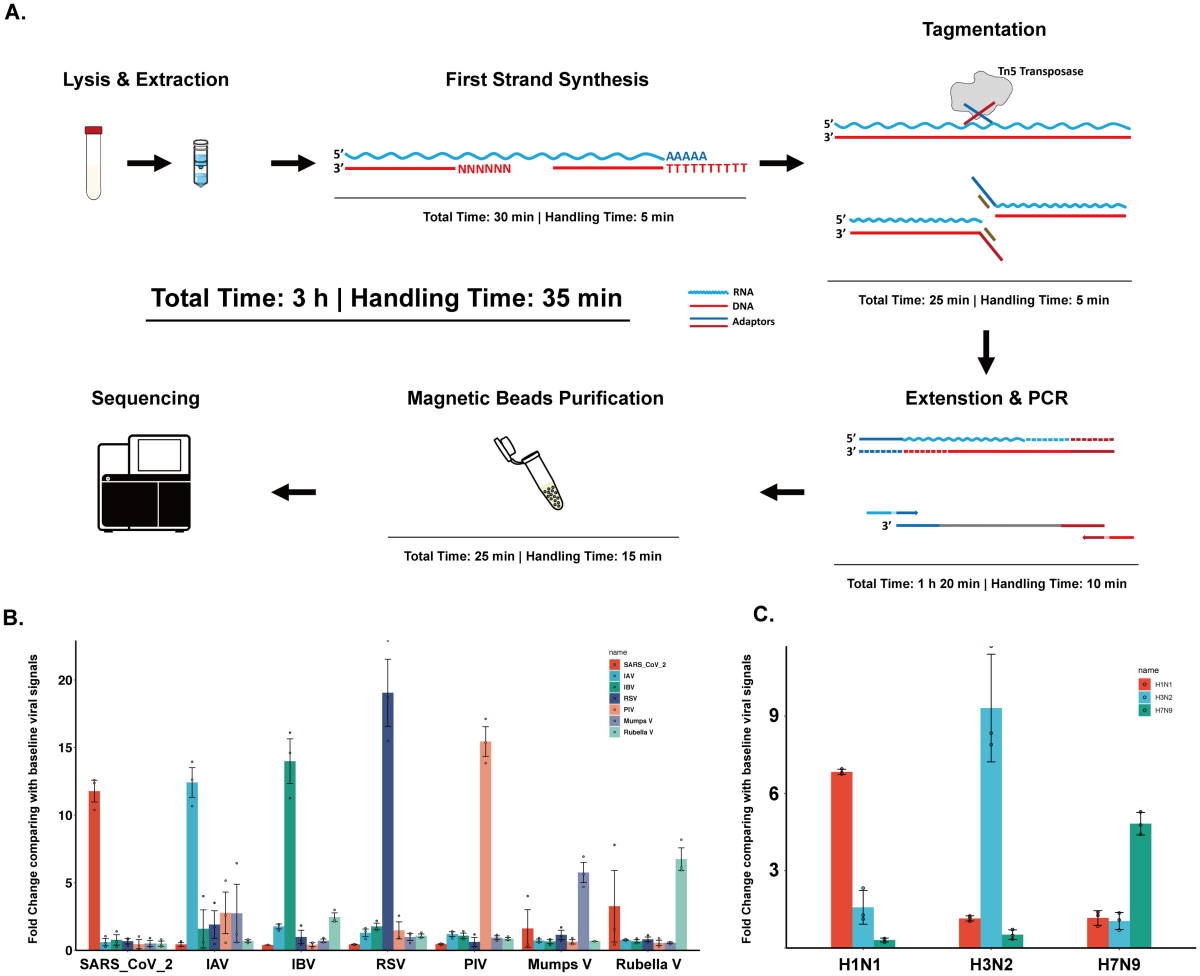
Overview of the optimized CATCH workflow and semiquantitative detection of respiratory RNA viruses. **(A)** Schematic overview of the RNA/DNA hybrid tagmentation-based mNGS workflow for respiratory RNA virus detection. The workflow includes sample lysis and extraction, first-strand synthesis, direct Tn5-based tagmentation of RNA/DNA hybrids, extension and indexed PCR amplification, magnetic bead purification, and sequencing. **(B)** Fold changes in duplicate-filtered reads per million (RPM) for seven respiratory RNA viruses after a tenfold increase in viral input. **(C)** Fold changes in duplicate-filtered RPM for influenza A virus subtypes A/2009/H1N1, A/H3N2, and A/H7N9 after a tenfold increase in viral input. Error bars indicate variation among replicate experiments.

To improve the clinical deployability of metagenomic sequencing for respiratory RNA virus detection, we optimized the CATCH workflow to reduce turnaround time and hands-on complexity. By streamlining key enzymatic steps and consolidating library preparation, the total library construction time was shortened to approximately 3 h, with about 35 min of hands-on time, enabling same-day sequencing-based testing in routine laboratory settings (Supplementary material; Figure 1A).

The optimized workflow was intended to improve clinical usability rather than introduce a new tagmentation chemistry. Compared with the earlier feasibility-oriented CATCH workflow, the current protocol reduced hands-on complexity and was evaluated through a more comprehensive validation framework, including LOD estimation, reproducibility testing, short-term stability assessment, host-cell interference analysis, semiquantitative evaluation, and clinical comparison with RT-qPCR or multiplex PCR assays. In the retrospective SARS-CoV-2 cohort, the optimized workflow showed a sensitivity of 84.5% and a specificity of 100% compared with clinical RT-qPCR results; sensitivity increased to 91.4% when the analysis was restricted to dual-gene-positive samples. In the prospective respiratory cohort, the overall sensitivity was 91.7% compared with routine multiplex PCR testing.

Using this streamlined workflow, we evaluated CATCH against a panel of clinically relevant respiratory RNA viruses, including SARS-CoV-2, influenza A virus (A/2009/H1N1), influenza B virus (B/Victoria), respiratory syncytial virus (RSV), parainfluenza virus, mumps rubulavirus, and rubella virus. All seven viruses were consistently detected at baseline input levels, indicating that workflow simplification preserved broad compatibility across phylogenetically diverse respiratory RNA viruses (Figure 1B; Table 1).

**Table 1:** Detection and quantification performance of 7 RNA viruses and 3 influenza A virus subtypes by CATCH.

Viral name	Genome size	GC%	1× Duplicate filtered RPM	10× Duplicate filtered RPM	Fold-change comparing with baseline (1×)	*p*-value
SARS-CoV-2	3 k	38.0	20.15 (3.02, 37.28)	215.12 (160.07, 270.16)	11.78 (8.76, 14.79)	0.126
Influenza A virus [A/2009(H1N1)]	13 k	44.2	177.06 (141.03, 213.09)	2198.24 (1471.33, 2925.15)	12.41 (8.31, 16.52)	0.127
Influenza B virus (B/Victoria)	14 k	40.2	9.08 (5.53, 12.62)	126.99 (71.07, 182.92)	13.99 (7.83,20.15)	0.108
Respiratory syncytial virus	15 k	33.2	11.22 (7.55, 14.88)	213.73 (110.10, 317.36)	19.05 (9.82, 28.29)	0.051
Parainfluenza virus	15 k	37.2	12.37 (5.84, 18.89)	191.04 (140.71, 241.37)	15.45 (11.38, 19.52)	0.029
Rubella virus	9.7 k	69.6	17.76 (12.37, 23.16)	119.89 (6.49, 17.49)	6.75 (3.65, 9.85)	0.046
Mumps rubulavirus	15 k	42.5	20.05 (8.38, 31.77)	165.39 (59.58, 171.92)	5.76 (2.96, 8.55)	0.022
Influenza A virus [A/2009(H1N1)]	13 k	44.2	4.73 (3.90, 5.55)	16.49 (10.46, 22.52)	3.36 (2.13, 4.59)	0.002
Influenza A virus (A/H3N2)	13 k	45.0	5.25 (2.95, 7.55)	38.92 (11.26, 66.58)	8.09 (2.34, 13.84)	0.29
Influenza A virus (A/H7N9)	13 k	45.1	1.10 (0.62, 1.57)	5.36 (3.08, 7.63)	1.69 (0.97, 2.40)	<0.001

### CATCH detected diverse respiratory RNA viruses and showed semiquantitative trends

To assess semiquantitative performance, viral input was increased tenfold relative to baseline, and changes in duplicate-filtered RPM were evaluated. Overall, sequencing-derived signals correlated with input concentration (Spearman *r* = 0.60, *p* < 1 × 10^−5^), supporting the use of RPM as an indicator of viral abundance trends. However, fold changes varied across viruses. RSV and parainfluenza virus showed stronger-than-expected increases, whereas mumps rubulavirus and rubella virus showed attenuated responses relative to the nominal tenfold input increase. These differences likely reflect virus-specific genomic and biochemical features, such as genome structure, GC content, RNA secondary structure, and reverse-transcription efficiency, rather than assay instability (Figure 1B; Table 1).

Given the clinical importance of influenza A virus subtyping, we further evaluated the ability of CATCH to distinguish and semiquantify closely related influenza A subtypes, including A/2009/H1N1, A/H3N2, and A/H7N9. All three subtypes were reliably detected; however, subtype-specific fold-change responses differed. A/H3N2 showed near-proportional signal scaling, whereas A/2009/H1N1 and A/H7N9 showed attenuated responses relative to input concentration (Figure 1C; Table 1).

Together, these results indicate that the CATCH workflow enables robust detection of a broad range of respiratory RNA viruses while providing biologically informative semiquantitative signals. At the same time, the observed virus- and subtype-specific variability highlights inherent constraints on absolute quantification in RNA virus mNGS and underscores the need for context-aware interpretation of sequencing-derived abundance metrics (Tan et al., 2024; Zhu et al., 2025).

### CATCH showed clinically relevant analytical sensitivity and robustness for SARS-CoV-2 and influenza a virus

To define the analytical performance of the optimized CATCH workflow in clinically relevant settings, we selected SARS-CoV-2 and influenza A virus as representative respiratory RNA viruses for in-depth evaluation. These viruses differ in genomic architecture and clinical testing context and together represent major use cases for respiratory virus diagnostics.

Using respiratory sample matrices spiked with serial dilutions of reference standards, CATCH showed a monotonic relationship between viral input concentration and sequencing-derived abundance for both SARS-CoV-2 and influenza A virus across the tested dilution ranges (Supplementary Figures S1A,B). Duplicate-filtered RPM values decreased with decreasing viral input, supporting the suitability of the sequencing signal as a semiquantitative surrogate of viral burden under controlled conditions.

Based on probit regression analysis, the 95% limits of detection for SARS-CoV-2 and influenza A virus were within clinically actionable ranges (Table 2). At concentrations near the estimated LODs, viral targets were detected with high reproducibility across replicate experiments. These findings indicate that the streamlined CATCH workflow preserved analytical sensitivity despite the substantial reduction in library preparation time (CLSI, 2012).

**Table 2:** Performance characteristics for CATCH on influenza A virus and SARS-CoV-2.

Performance metrics	Method	Results
Limits of detection	SARS-CoV-2	39.2 copies/test
	Influenza A virus (A/H1N1/2009)	278.1 copies/mL
Precision	Qualitative detection of both viruses over 5 consecutive runs	100% concordance
	Qualitative detection of both viruses over 3 replicates on the same runs	100% concordance
Stability	Qualitative detection of Influenza A virus held at 4 °C for 0, 3, 6 days	100% concordance
Interference	Qualitative detection of Influenza A virus in Spiked host cell A549 (low, medium and high concentration)	Only in 104 cell/mL, all spiked ICs are detected above QC thresholds; All spiked Influenza A virus are detected above QC expect for 2 replicates at high concentration level

To evaluate host-background interference, increasing concentrations of A549 human cells were introduced into spiked samples. High host-cell loads, particularly at or above 10^5^ cells/mL, markedly reduced both viral and internal control signals, consistent with the known limitations of mNGS-based assays in specimens with high host content (Supplementary Figure S1C; Table 2) (Gao et al., 2025; Fierer et al., 2025). On this basis, samples with an estimated host-cell concentration below 10^5^ cells/mL are preferred for direct CATCH processing. Samples with cellularity at or above this level should be flagged before library preparation, and additional preprocessing, such as mucus removal, low-speed clarification, dilution, host-cell depletion, or repeat sampling when clinically feasible, should be considered. Internal RNA control recovery and the proportion of host-derived reads should also be used as run-level QC indicators. This threshold should be interpreted as a preliminary operational recommendation derived from the present experimental system rather than as a universal rejection criterion.

Assay reproducibility and robustness were further evaluated through inter-run precision testing, intra-run replicate analysis, and short-term stability assessment. Qualitative detection was fully concordant across five consecutive runs and across triplicate replicates within the same run. In addition, influenza A virus remained qualitatively detectable after storage at 4 °C for up to 6 days, supporting the robustness of the CATCH workflow under routine laboratory conditions (Supplementary Figure S1D; Table 2).

Overall, these data establish the analytical performance boundaries of the optimized CATCH workflow for clinically important respiratory RNA viruses and demonstrate a practical balance between shortened turnaround time, analytical sensitivity, and assay robustness.

Clinical validation and added diagnostic value of CATCH in respiratory infection cohorts.

**Figure 2:**
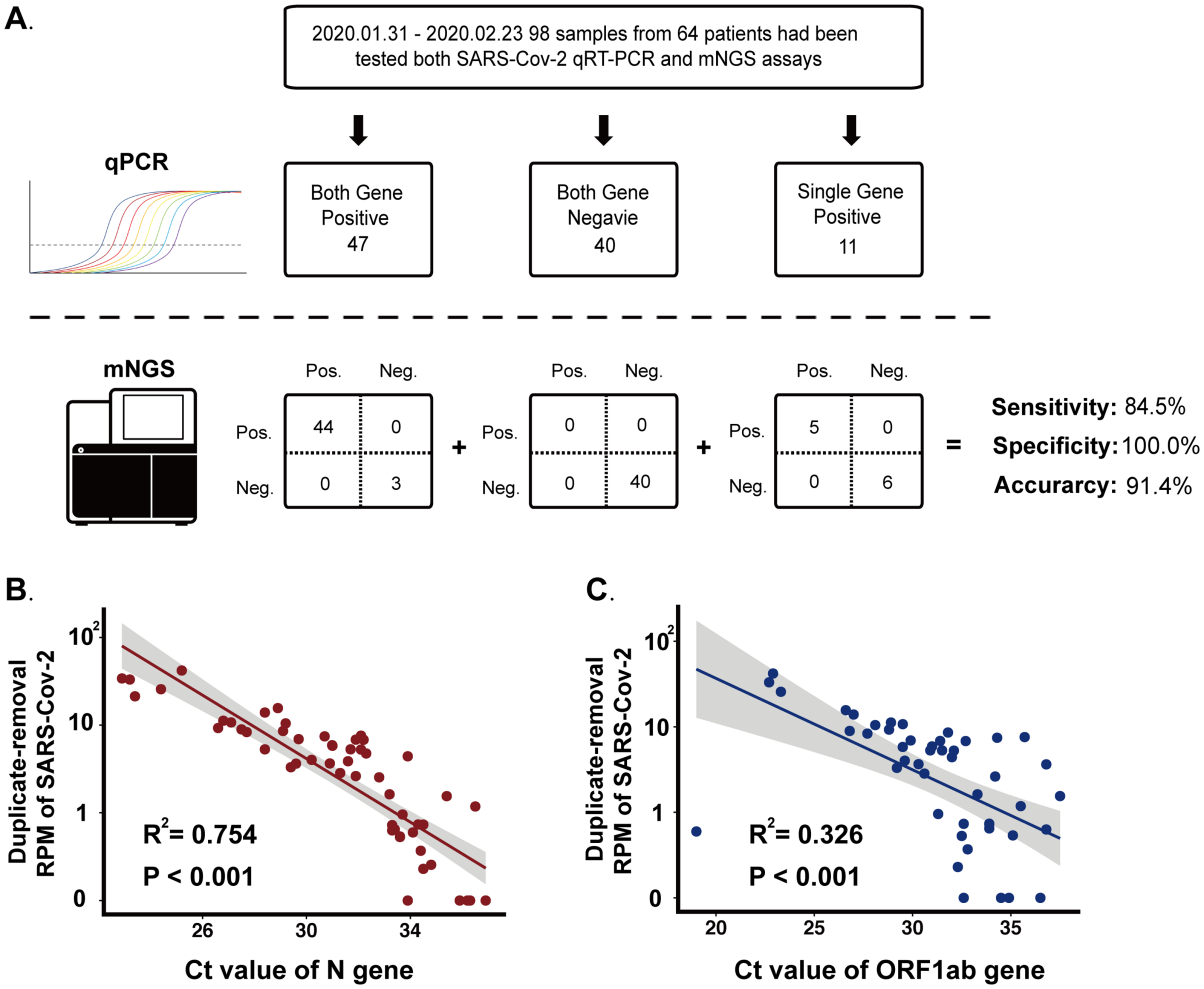
Clinical validation of CATCH for SARS-CoV-2 detection in respiratory samples. **(A)** Concordance between clinical RT-qPCR and CATCH mNGS results in 98 retrospective respiratory samples, including dual-gene-positive, dual-gene-negative, and single-gene-positive groups. **(B)** Correlation between RT-qPCR Ct values for the SARS-CoV-2N gene and duplicate-filtered SARS-CoV-2 RPM. **(C)** Correlation between RT-qPCR Ct values for ORF1ab and duplicate-filtered SARS-CoV-2 RPM.

To evaluate clinical performance, we applied the optimized CATCH workflow to both retrospective and prospective respiratory infection cohorts and benchmarked its diagnostic performance against routine molecular assays (Supplementary Table S2). In the retrospective cohort, 98 residual RNA samples from 64 patients with suspected COVID-19 were analyzed. Compared with clinical RT-qPCR results, CATCH achieved a sensitivity of 84.5% and a specificity of 100% for SARS-CoV-2 detection (Figure 2A). Sensitivity increased to 91.4% when the analysis was restricted to dual-gene-positive samples, indicating stronger performance in samples with higher viral burden. No SARS-CoV-2 reads or other respiratory viruses were detected in PCR-negative samples, supporting the high specificity of the assay.

Sequencing-derived SARS-CoV-2 abundance was strongly inversely correlated with RT-qPCR Ct values for the N gene (*R*^2^ = 0.754), whereas a weaker correlation was observed for ORF1ab (*R*^2^ = 0.326) (Figures 2B,C). This difference is biologically and technically plausible. The N gene is located near the 3′ end of the SARS-CoV-2 genome and is also represented in abundant subgenomic RNAs, whereas ORF1ab is located closer to the 5′ region. Because RNA/DNA hybrid tagmentation shows a detectable 3′ bias, reads from 3′-proximal regions may be recovered more consistently than reads from 5′ regions, particularly in samples with low viral abundance or partially degraded RNA. Differences in qPCR target design and amplification efficiency may further contribute to gene-specific differences in correlation. Therefore, CATCH-derived RPM should be interpreted as a semiquantitative indicator of viral burden rather than as a direct substitute for gene-specific qPCR Ct values.

In the prospective cohort, 63 respiratory samples collected between November 2023 and February 2024 were analyzed in parallel using CATCH and multiplex PCR assays. Among the 36 samples positive by routine molecular testing, CATCH correctly identified 33, corresponding to an overall sensitivity of 91.7%. Detected pathogens included SARS-CoV-2, influenza A virus, RSV, human parainfluenza virus, hMPV, and *Mycoplasma pneumoniae*, reflecting the broad pathogen coverage of the assay.

CATCH identified mixed respiratory infections in 12.1% (4/33) of positive samples in the prospective cohort, including both dual and triple viral combinations. Two of these coinfections were fully concordant with multiplex PCR results, whereas the remaining cases revealed additional viral pathogens not detected by the initial targeted assays and subsequently confirmed by follow-up PCR testing. Across both cohorts, CATCH showed high concordance with RT-qPCR and multiplex PCR while providing additional diagnostic information beyond predefined targeted testing (Tan et al., 2024; Gauthier et al., 2024; de Hoog et al., 2025; Hauser-van Westrhenen et al., 2026).

Collectively, these clinical validation results indicate that CATCH delivers diagnostic accuracy comparable to established molecular assays while offering broader pathogen detection and improved recognition of mixed infections, supporting its value as a complementary tool in complex respiratory infection diagnostics.

### Untargeted CATCH data also captured exploratory respiratory microbial community shifts

Leveraging the untargeted nature of the CATCH workflow, we further explored respiratory microbial community profiles using the same sequencing datasets generated for viral detection. This analysis was intended to assess the feasibility of extracting additional ecological information beyond targeted pathogen identification.

Comparative analysis of oropharyngeal swab samples from patients with COVID-19 and healthy controls revealed distinct microbial community structures, as shown by genus-level relative abundance and Jensen-Shannon divergence metrics (Supplementary Figures S2A,B). Compared with healthy controls, COVID-19 samples showed significantly altered microbial composition (PERMANOVA, *p* < 0.001), with further separation observed between critically ill and moderately ill patients (PERMANOVA, *p* = 0.013) (Quinn-Bohmann et al., 2024; Spottiswoode et al., 2024; von Ameln Lovison et al., 2025).

Several commensal genera commonly associated with a healthy upper respiratory tract, including Streptococcus, Prevotella, and Veillonella, were enriched in healthy controls, whereas samples from COVID-19 patients showed increased representation of opportunistic taxa. Candida was significantly enriched in critically ill patients (Mann–Whitney *U* test, *p* < 0.001; Supplementary Figure S2C), and culture-based testing using the VITEK2 system confirmed Candida positivity in a subset of ICU samples. This observation supports the biological plausibility of the sequencing signal but should be interpreted as an exploratory association rather than evidence of causality. Increased Candida abundance in critically ill patients may reflect ICU-related factors, such as intubation, antibiotic exposure, altered airway ecology, impaired mucosal clearance, and prolonged hospitalization.

Taken together, these exploratory findings illustrate that the CATCH workflow can simultaneously support respiratory virus detection and microbial community profiling within a single sequencing assay. Although not intended for definitive microbiome characterization, this dual-purpose capability highlights an added dimension of untargeted mNGS that is not available with conventional PCR-based diagnostics. Further prospective studies with standardized sampling, contamination control, and detailed clinical metadata are needed to determine the clinical relevance of these microbial community signals (Quinn-Bohmann et al., 2024; Spottiswoode et al., 2024; von Ameln Lovison et al., 2025).

## Discussion

In the post-pandemic era, routine respiratory diagnostics increasingly require methods that can detect diverse viral pathogens, identify mixed infections, and remain feasible for clinical implementation, a need also reflected in recent evaluations of respiratory pathogen sequencing workflows (Tan et al., 2024; Gauthier et al., 2024; Yin et al., 2024). In this study, we optimized and clinically validated CATCH, a rapid RNA/DNA hybrid tagmentation-based mNGS workflow for respiratory RNA virus detection. By substantially reducing library preparation time while preserving analytical sensitivity and specificity, CATCH addresses a practical diagnostic gap between targeted PCR assays and conventional metagenomic sequencing. These findings support its use as a clinically deployable sequencing-based approach for timely pathogen detection, coinfection identification, and semiquantitative viral assessment in real-world respiratory infection settings (Gaston, 2023; Tan et al., 2024; Gauthier et al., 2024; Alcolea-Medina et al., 2025). It should be noted that this study did not include a direct head-to-head comparison between the previously reported and optimized CATCH workflows using the same clinical specimens. Therefore, the reported sensitivity and specificity should be interpreted as the clinical performance of the optimized workflow in the current validation cohorts, rather than as evidence of diagnostic superiority over the earlier protocol. The main advance of the present study lies in workflow simplification, reduced library preparation time, and systematic analytical and clinical validation in respiratory infection settings.

A major strength of CATCH is its ability to detect a broad range of respiratory RNA viruses within a streamlined and time-efficient protocol. By consolidating reverse transcription, hybrid tagmentation, and indexed amplification steps, we reduced total library preparation time to approximately 3 h with limited hands-on input. This turnaround time approaches that of advanced molecular panel workflows while retaining the hypothesis-free nature of mNGS, supporting the practical use of CATCH in clinical laboratories facing increasingly complex respiratory pathogen landscapes. The optimized workflow showed robust analytical performance across multiple viral species, including SARS-CoV-2, influenza A/B virus, RSV, hMPV, and parainfluenza viruses, with high specificity and reproducibility.

Beyond qualitative detection, this study highlights both the semiquantitative potential and the biological constraints of RNA virus mNGS based on hybrid tagmentation. Duplicate-filtered RPM values correlated with viral input concentration across dilution experiments, supporting the use of CATCH-derived sequencing signals as indicators of viral burden trends. However, deviations from the expected tenfold signal increase were observed for several viruses and influenza A subtypes. These differences likely reflect virus-specific genomic and biochemical features, including RNA secondary structure, genome segmentation, GC content, and reverse-transcription efficiency, rather than random assay variability. Thus, although mNGS-based approaches can provide biologically meaningful semiquantitative information, absolute quantification across diverse RNA viruses remains inherently constrained. This observation defines a realistic performance boundary for RNA virus mNGS and highlights the need for virus-aware interpretation of sequencing-derived abundance metrics (Tan et al., 2024; Zhu et al., 2025).

The 3′ bias associated with RNA/DNA hybrid tagmentation may also affect genome-wide coverage uniformity and should be considered when sequencing data are used for variant or lineage interpretation. This bias is unlikely to compromise qualitative detection of SARS-CoV-2 or influenza virus when sufficient viral reads are present, because pathogen identification in this study was based on species-level read assignment and multiple nonoverlapping reads. However, comprehensive variant or lineage assignment may be affected if lineage-defining mutations are located in poorly covered 5′ regions or genomic segments. Therefore, CATCH should be regarded primarily as a diagnostic detection workflow in the present study. Lineage assignment or mutation analysis should only be attempted when coverage across relevant genomic regions is adequate, and targeted PCR, amplicon sequencing, or deeper sequencing should be considered when key regions are insufficiently covered.

Clinical validation in retrospective and prospective cohorts further supports the diagnostic utility of CATCH. Compared with RT-qPCR, the assay showed high sensitivity and 100% specificity for SARS-CoV-2 detection, with improved sensitivity when the analysis was restricted to dual-gene-positive samples. The strong inverse correlation between SARS-CoV-2 RPM values and N-gene Ct values suggests that CATCH-derived sequencing signals reflect underlying viral burden, although with a detectable 3′ bias. This bias is relevant to quantitative interpretation but did not appear to compromise qualitative detection in the present cohorts. CATCH also identified mixed respiratory infections, including cases initially missed by targeted PCR panels and subsequently confirmed by follow-up testing. This ability to detect unexpected coinfections may be particularly valuable in immunocompromised patients and in severe pneumonia, where pathogen spectra are often broad and dynamic (de Hoog et al., 2025; Hauser-van Westrhenen et al., 2026; Lim et al., 2016). Nevertheless, additional viral detections require cautious clinical interpretation. Mixed infections detected by CATCH may represent clinically relevant coinfections, prolonged viral shedding, or incidental low-level detection, depending on the clinical context. In this study, several additional detections were confirmed by follow-up targeted PCR, supporting their analytical validity. However, the number of mixed infection cases was small, and the study was not powered to determine whether mixed infection was independently associated with clinical severity. Therefore, CATCH results should be interpreted together with symptoms, sampling time, viral RPM, confirmatory qPCR results when available, immune status, radiological findings, and disease course.

Host background remains a recognized challenge for mNGS-based diagnostics, and our findings show that high concentrations of human cells (>10^5^ cells/mL) can markedly reduce both viral and internal control signals. This observation is consistent with prior studies and highlights the importance of appropriate specimen selection and preprocessing, particularly for lower respiratory tract samples (Gao et al., 2025; Fierer et al., 2025). Samples with high host-cell content may require dilution, mucus removal, low-speed clarification, host-cell depletion, repeat sampling, or deeper sequencing, depending on clinical feasibility. Internal control recovery and the proportion of host-derived reads should also be considered when interpreting negative results. Despite this limitation, the reproducibility and short-term stability observed across multiple runs and storage conditions support the robustness of the CATCH workflow under routine laboratory conditions.

In addition to pathogen detection, we explored the broader respiratory microbial landscape using the same CATCH-derived sequencing data. This analysis should be regarded as exploratory. The enrichment of opportunistic taxa such as Candida in critically ill patients may reflect critical illness and ICU-related exposures, including intubation, antibiotic use, altered airway ecology, impaired mucosal clearance, and prolonged hospitalization, rather than a causal contribution to disease susceptibility or progression. Culture-based testing partially supported this sequencing-based observation, but the current data are insufficient to determine whether Candida contributed to disease progression or simply reflected critical illness and ICU care. Nevertheless, these findings illustrate a potential added value of untargeted sequencing: the same dataset may provide pathogen detection together with broader microbial ecological context. This capability is not available with conventional PCR-based diagnostics and may help generate hypotheses regarding secondary infection risk and host–microbe interactions. Dedicated prospective studies with standardized sampling, contamination control, and detailed clinical metadata are needed before microbiome-derived signals can be used for clinical decision-making (Quinn-Bohmann et al., 2024; Spottiswoode et al., 2024; von Ameln Lovison et al., 2025).

This study has several limitations. First, CATCH provides semiquantitative rather than absolute quantitative information. Although duplicate-filtered RPM can reflect viral abundance trends under comparable experimental conditions, it should not be interpreted as an absolute viral load without virus-specific calibration. Second, assay performance may be affected by sample biomass, RNA integrity, viral abundance, and host nucleic acid background. In particular, samples with excessive host-cell content may show reduced viral and internal control signals, thereby increasing the risk of false-negative results. Third, semiquantitative performance differed among viral species, indicating that virus-specific biases need to be further characterized and that appropriate normalization strategies should be developed. Fourth, although the clinical validation cohorts included a range of respiratory pathogens, larger multicenter studies are required to establish diagnostic performance across broader patient populations and specimen types. In addition, because the archived retrospective SARS-CoV-2 dataset did not retain a complete per-sample continuous RPM table suitable for threshold-based ROC analysis, we did not perform a *post hoc* ROC-based cut-off analysis; diagnostic performance was therefore assessed using predefined positivity criteria and 2 × 2 agreement analysis. Finally, the microbiome-related findings should be interpreted with caution because of the limited sample size, possible batch effects, and the risk of contamination in low-biomass samples. Dedicated prospective studies will be needed to confirm these exploratory observations (Fierer et al., 2025; Salter et al., 2014).

In conclusion, CATCH is a rapid and clinically deployable RNA virus mNGS workflow that helps bridge targeted molecular diagnostics and conventional metagenomic sequencing. By combining broad pathogen detection, coinfection identification, and semiquantitative assessment within a streamlined protocol, CATCH provides a practical framework for modern respiratory infection diagnostics and syndromic surveillance. Further integration with automated laboratory pipelines and prospective outcome-based studies may help define its role in precision infectious disease management.

## Data Availability

Due to historical data-retention limitations for early-stage retrospective sequencing runs, a subset of raw FASTQ files could not be retrieved. The host-depleted RNA metagenomic sequencing data that remain available for the prospective validation cohort have been deposited in the Genome Sequence Archive (GSA) of the National Genomics Data Center (NGDC) under accession number CRA045316. The data are available at: https://ngdc.cncb.ac.cn/gsa/browse/CRA045316.

## References

[ref1] Alcolea-Medina A. Alder C. Snell L. B. Charalampous T. Aydin A. Nebbia G. . (2025). Rapid pan-microbial metagenomics for pathogen detection and personalised therapy in the intensive care unit: a single-Centre prospective observational study. Lancet Microbe 6:101174. doi: 10.1016/j.lanmic.2025.101174 41045941

[ref2] Bao Y. Bolotov P. Dernovoy D. Kiryutin B. Zaslavsky L. Tatusova T. . (2008). The influenza virus resource at the National Center for biotechnology information. J. Virol. 82, 596–601. doi: 10.1128/JVI.02005-07, 17942553 PMC2224563

[ref3] Camacho C. Coulouris G. Avagyan V. Ma N. Papadopoulos J. Bealer K. . (2009). BLAST+: architecture and applications. BMC Bioinformatics 10:421. doi: 10.1186/1471-2105-10-421, 20003500 PMC2803857

[ref4] Carter L. L. Yu M. A. Sacks J. A. Barnadas C. Pereyaslov D. Cognat S. . (2022). Global genomic surveillance strategy for pathogens with pandemic and epidemic potential 2022–2032. Bull. World Health Organ. 100, 239–239A. doi: 10.2471/BLT.22.288220, 35386562 PMC8958828

[ref5] Chen H. Huang Q. Wu W. Wang Z. Wang W. Liu Y. . (2024). Assessment and clinical utility of metagenomic next-generation sequencing for suspected lower respiratory tract infections. Eur. J. Med. Res. 29:213. doi: 10.1186/s40001-024-01806-7, 38561853 PMC10983704

[ref6] Chen S. Zhou Y. Chen Y. Gu J. (2018). Fastp: an ultra-fast all-in-one FASTQ preprocessor. Bioinformatics 34, i884–i890. doi: 10.1093/bioinformatics/bty560, 30423086 PMC6129281

[ref7] Chiu C. Y. Miller S. A. (2019). Clinical metagenomics. Nat. Rev. Genet. 20, 341–355. doi: 10.1038/s41576-019-0113-7, 30918369 PMC6858796

[ref8] Clarke E. L. Taylor L. J. Zhao C. Connell A. Lee J. J. Fett B. . (2019). Sunbeam: an extensible pipeline for analyzing metagenomic sequencing experiments. Microbiome 7:46. doi: 10.1186/s40168-019-0658-x, 30902113 PMC6429786

[ref9] Clinical and Laboratory Standards Institute (CLSI) (2012). Evaluation of Detection Capability for Clinical Laboratory Measurement Procedures. 2nd Edn. CLSI guideline EP17-A2 Wayne: Clinical and Laboratory Standards Institute.

[ref10] de Hoog M. L. A. Hauser-van Westrhenen E. S. E. M. Winkel A. M. A. M. de Jong M. D. van Houten M. A. Lelyveld S. F. L. . (2025). Impact of co-infection with SARS-CoV-2 and other respiratory viruses on illness: pooled analyses of 11 COVID-19 cohorts. J. Infect. 90:106501. doi: 10.1016/j.jinf.2025.106501, 40349729

[ref11] Di L. Fu Y. Sun Y. Li J. Liu L. Yao J. . (2020). RNA sequencing by direct tagmentation of RNA/DNA hybrids. Proc. Natl. Acad. Sci. USA 117, 2886–2893. doi: 10.1073/pnas.1919800117, 31988135 PMC7022195

[ref12] Fierer N. Leung P. M. Lappan R. Eisenhofer R. Ricci F. Holland S. I. . (2025). Guidelines for preventing and reporting contamination in low-biomass microbiome studies. Nat. Microbiol. 10, 1570–1580. doi: 10.1038/s41564-025-02035-2, 40542287

[ref13] Gao Y. Luo H. Lyu H. Yang H. Yousuf S. Huang S. . (2025). Benchmarking short-read metagenomics tools for removing host contamination. Gigascience 14:giaf004. doi: 10.1093/gigascience/giaf004, 40036691 PMC11878760

[ref14] Gaston D. C. (2023). Clinical metagenomics for infectious diseases: progress toward operational value. J. Clin. Microbiol. 61:e01267-22. doi: 10.1128/jcm.01267-22, 36728425 PMC9945490

[ref15] Gauthier N. P. G. Chan W. Locher K. Smailus D. Coope R. Charles M. . (2024). Validation of an automated, end-to-end metagenomic sequencing assay for agnostic detection of respiratory viruses. J. Infect. Dis. 230, e1245–e1253. doi: 10.1093/infdis/jiae226, 38696336 PMC11646614

[ref16] Hauser-van Westrhenen E. Junquera Guinovart L. Schuurman R. van Boven M. Bonten M. Bruijning-Verhagen P. (2026). Associations between virus single infection or coinfection and respiratory symptoms in young children: a community-based cohort study. Int. J. Infect. Dis. 164:108317. doi: 10.1016/j.ijid.2025.108317, 41422943

[ref17] Jin X. Li J. Shao M. Li C. Zhang B. Zhang Y. . (2022). Improving suspected pulmonary infection diagnosis by bronchoalveolar lavage fluid metagenomic next-generation sequencing: a multicenter retrospective study. Microbiol. Spectrum 10:e02473-21. doi: 10.1128/spectrum.02473-21, 35943274 PMC9431624

[ref18] Kopylova E. Noé L. Touzet H. (2012). SortMeRNA: fast and accurate filtering of ribosomal RNAs in metatranscriptomic data. Bioinformatics 28, 3211–3217. doi: 10.1093/bioinformatics/bts611, 23071270

[ref19] Lim F. J. de Klerk N. Blyth C. C. Fathima P. Moore H. C. (2016). Systematic review and meta-analysis of respiratory viral coinfections in children. Respirology 21, 648–655. doi: 10.1111/resp.12741, 26919484

[ref20] Lu B. Dong L. Yi D. Zhang M. Zhu C. Li X. . (2020). Transposase-assisted tagmentation of RNA/DNA hybrid duplexes. eLife 9:e54919. doi: 10.7554/eLife.54919, 32701057 PMC7402673

[ref21] Quinn-Bohmann N. Freixas-Coutin J. A. Seo J. Simmons R. Diener C. Gibbons S. M. (2024). Meta-analysis of the human upper respiratory tract microbiome reveals robust taxonomic associations with health and disease. BMC Biol. 22:93. doi: 10.1186/s12915-024-01887-0, 38654335 PMC11040984

[ref22] Salter S. J. Cox M. J. Turek E. M. Calus S. T. Cookson W. O. Moffatt M. F. . (2014). Reagent and laboratory contamination can critically impact sequence-based microbiome analyses. BMC Biol. 12:87. doi: 10.1186/s12915-014-0087-z, 25387460 PMC4228153

[ref23] Schlaberg R. Chiu C. Y. Miller S. Procop G. W. Weinstock G. (2017). Validation of metagenomic next-generation sequencing tests for universal pathogen detection. Arch. Pathol. Lab Med. 141, 776–786. doi: 10.5858/arpa.2016-0539-RA, 28169558

[ref24] Spottiswoode N. Tsitsiklis A. Chu V. T. Phan H. V. DeVoe C. Love C. . (2024). Microbial dynamics and pulmonary immune responses in COVID-19 secondary bacterial pneumonia. Nat. Commun. 15:9339. doi: 10.1038/s41467-024-53566-x, 39472555 PMC11522429

[ref25] Tan J. K. Servellita V. Stryke D. Kelly E. Streithorst J. Sumimoto N. . (2024). Laboratory validation of a clinical metagenomic next-generation sequencing assay for respiratory virus detection and discovery. Nat. Commun. 15:9016. doi: 10.1038/s41467-024-51470-y, 39532844 PMC11558004

[ref26] von Ameln Lovison O. Marino S. F. Abdulle A. Khan M. Akbar A. Doughty E. L. . (2025). Unveiling the role of the upper respiratory tract microbiome in susceptibility and severity to COVID-19. Front. Cell. Infect. Microbiol. 15:1531084. doi: 10.3389/fcimb.2025.1531084, 40433668 PMC12106449

[ref27] Wei F. Yu Y. Hu Z. Wang R. Guo X. Jin H. . (2020). Laboratory validation of an RNA/DNA hybrid tagmentation based mNGS workflow on SARS-CoV-2 and other respiratory RNA viruses detection. medRxiv. doi: 10.1101/2020.05.12.20099754

[ref28] Wood D. E. Lu J. Langmead B. (2019). Improved metagenomic analysis with kraken 2. Genome Biol. 20:257. doi: 10.1186/s13059-019-1891-0, 31779668 PMC6883579

[ref29] World Health Organization (2024). Implementing the Integrated Sentinel Surveillance of Influenza and Other Respiratory Viruses of Epidemic and Pandemic Potential by the GISRS. Geneva: World Health Organization.

[ref30] World Health Organization (2025). Expanding the Global Influenza Surveillance and Response System to Include Other Respiratory Viruses with Epidemic or Pandemic Potential. Geneva: World Health Organization.

[ref31] Yang A. Chen C. Hu Y. Zheng G. Chen Z. Li H. . (2022). Application of metagenomic next-generation sequencing (mNGS) using bronchoalveolar lavage fluid (BALF) in diagnosing pneumonia of children. Microbiol. Spectrum 10:e01488-22. doi: 10.1128/spectrum.01488-22, 36169415 PMC9603332

[ref32] Yin Y. Zhu P. Guo Y. Li Y. Chen H. Liu J. . (2024). Enhancing lower respiratory tract infection diagnosis: implementation and clinical assessment of multiplex PCR-based and hybrid capture-based targeted next-generation sequencing. EBioMedicine 107:105307. doi: 10.1016/j.ebiom.2024.105307, 39226681 PMC11403251

[ref33] Zhang Z. Liu D. Wang D. Wu Q. (2021). Library preparation based on transposase-assisted RNA/DNA hybrid co-tagmentation for next-generation sequencing of human noroviruses. Viruses 13:65. doi: 10.3390/v13010065, 33418922 PMC7825083

[ref34] Zheng Y. Liu Y. Feng L. Wu J. Li X. Guo Q. (2024). Clinical utility of metagenomic next-generation sequencing on bronchoalveolar lavage fluid in diagnosis of lower respiratory tract infections. BMC Pulm. Med. 24:422. doi: 10.1186/s12890-024-03237-w, 39210307 PMC11360863

[ref35] Zhu T. Y. Guo J. Y. Zhang D. Weng L. Peng J. M. Yang Q. W. . (2025). A comparative study of DNA- and RNA-metagenomic next-generation sequencing for pathogen detection in lower respiratory tract infections. J. Infect. 91:106659. doi: 10.1016/j.jinf.2025.106659, 41270973

