# Supplementary material for: Rapid clinical validation of an RNA/DNA hybrid tagmentation-based metagenomic workflow for respiratory RNA virus detection
Source: Front Microbiol. 2026 Jul 15;17:1849991. doi: 10.3389/fmicb.2026.1849991 (PMC13416238; doi:10.3389/fmicb.2026.1849991)
Supplement: Supplementary file 1 [file Table_1.docx]

Supplementary information

1. Methods

Data processing

Duplicate-filtered reads per million (RPM) for each virus were calculated as follows: (1) reads assigned to target viral species (excluding SARS-CoV-2) were extracted; (2) reads were aligned to the corresponding reference genome using tblastx (-max_target_seqs 1 -max_hsps 1 -evalue 1e-5); reads with ≥90% identity and ≥90% query coverage were retained; (3) reads sharing the same start position were marked as PCR duplicates and removed; and (4) the remaining reads were normalized to the sequencing depth to obtain the RPM. A virus was considered positive if (i) ≥3 nonoverlapping reads mapped to distinct genomic regions and (ii) the duplicate-filtered RPM was ≥10-fold greater than that of the no-template control (NTC). Other microbial taxa were treated as background contaminants and excluded.

Study design of respiratory RNA viral species and subspecies detection by CATCH

Seven respiratory RNA viruses and three influenza A subtypes were evaluated. For viruses/subtypes without certified copy number standards, we tested the highest dilution level (approximating the LOD) as the baseline input. SARS-CoV-2 and influenza A virus (A/2009/H1N1) standards with known concentrations were also included; these concentrations were used to define baselines for the remaining six RNA viruses and two influenza A subtypes. For each virus or subtype, the input was increased tenfold relative to the baseline, and each condition was tested in triplicate. For SARS-CoV-2 and influenza A virus (A/2009/H1N1), the baseline inputs were 10^2^ copies/test and 10^4^ copies/mL, respectively.

1. Figures and tables


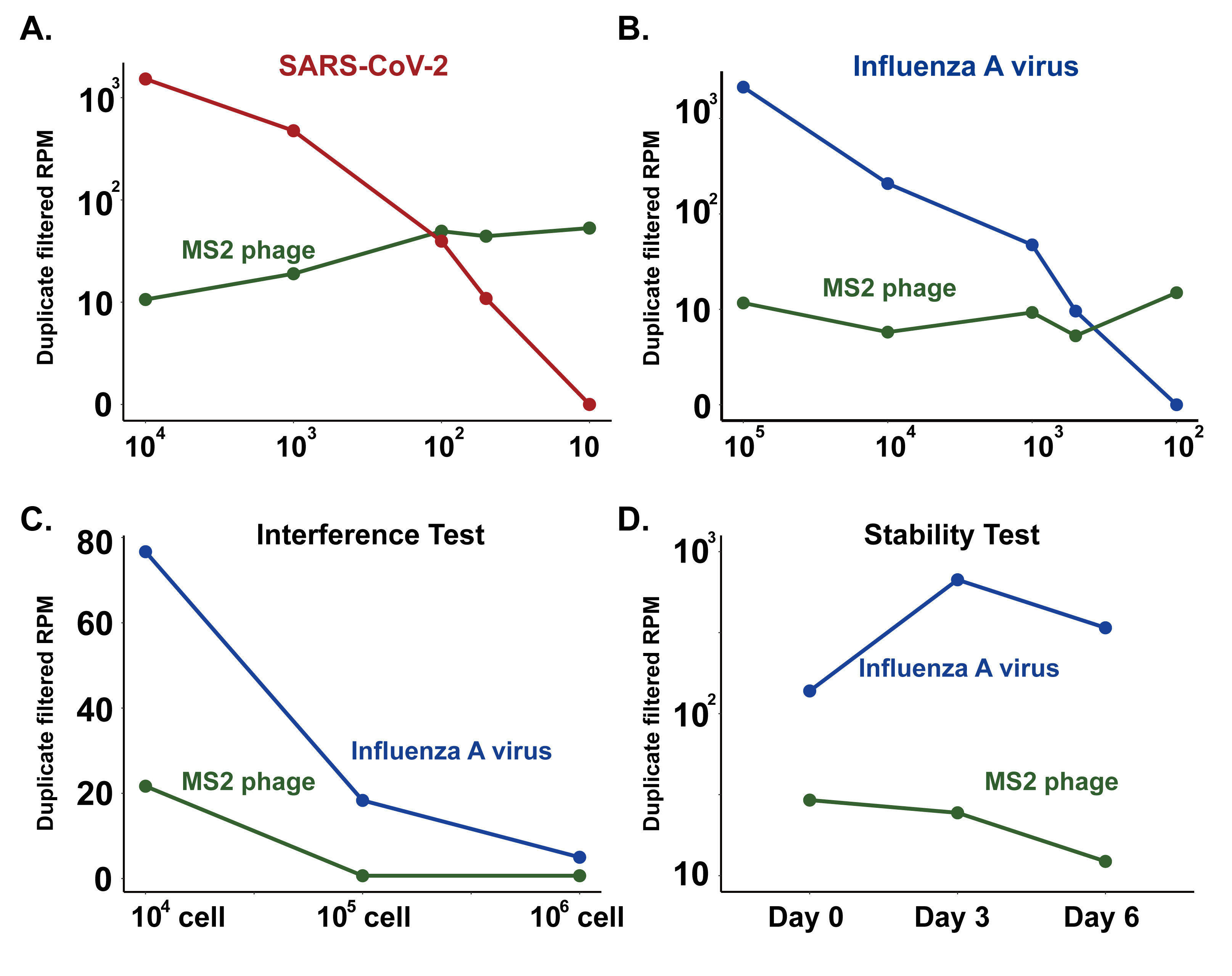


**Figure S1.** Analytical characteristics of SARS-CoV-2 and the influenza A virus.


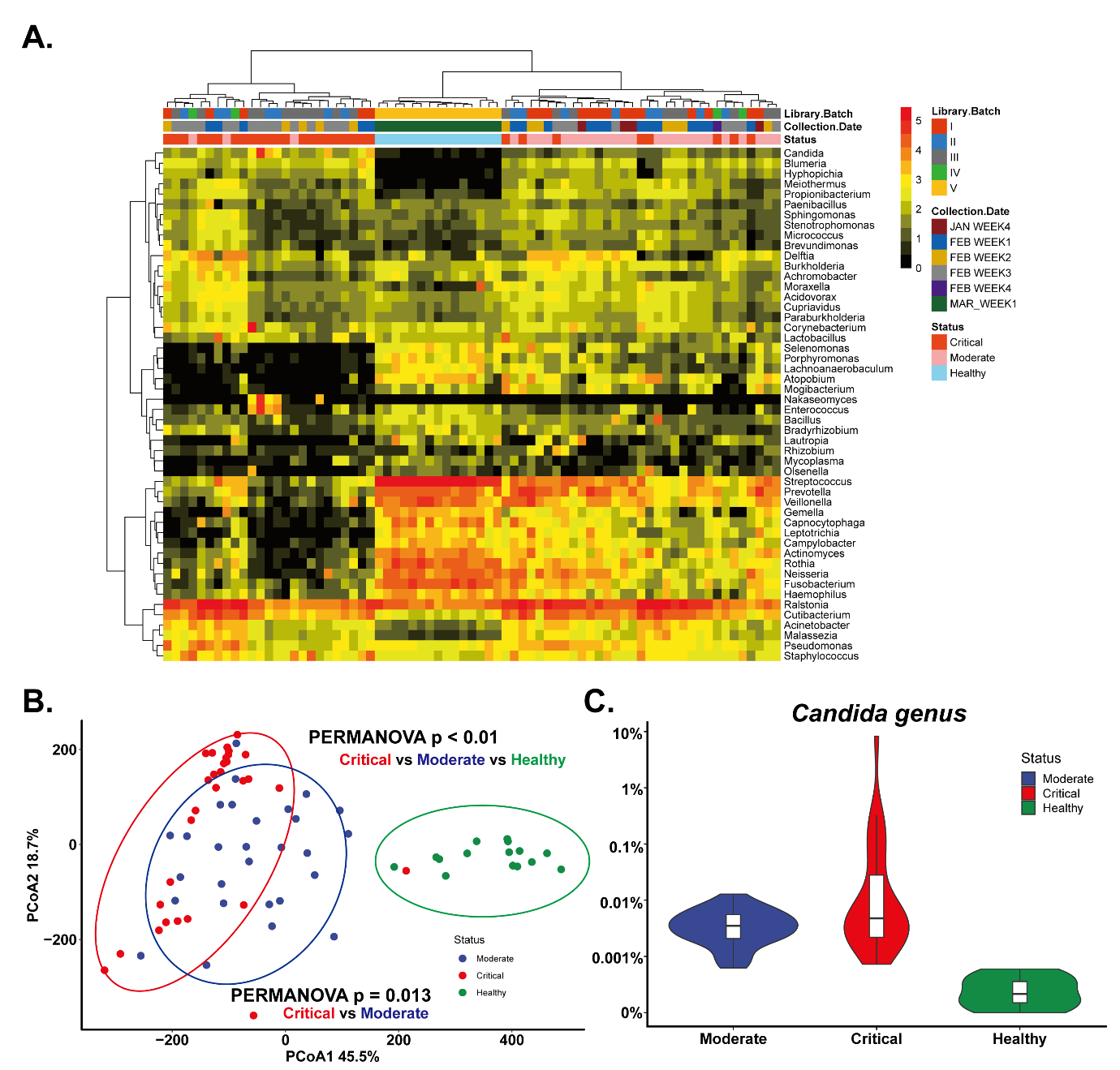


**Figure S2.** Microbial profiles of oropharyngeal swabs from critically ill patients, moderately ill patients and healthy volunteers

**Table S1. Comparison between the previously reported CATCH workflow and the optimized workflow used in this study**

| Item | Previous CATCH workflow | Optimized CATCH workflow in this study |
| --- | --- | --- |
| Study aim | Technical feasibility of RNA/DNA hybrid tagmentation for RNA virus sequencing | Clinical validation of a rapid workflow for respiratory RNA virus detection |
| Core principle | Tn5-based RNA/DNA hybrid tagmentation | Same principle, with workflow optimization for clinical use |
| Workflow time | Not optimized for rapid clinical testing | Approximately 3 h library preparation with about 35 min hands-on time |
| Reference method | Not systematically benchmarked in the current validation framework | Compared with RT-qPCR or multiplex PCR |
| Diagnostic interpretation | Focused on feasibility | Sensitivity, specificity, and concordance assessed in the present cohorts |
| Additional output | Viral detection | Viral detection, mixed infection identification, and exploratory microbial profiling |
| Main conclusion | Demonstrated feasibility of the CATCH strategy | Supports CATCH as a rapid, clinically deployable respiratory RNA virus mNGS workflow |

**Abbreviations:** CATCH, RNA/DNA hybrid tagmentation-based metagenomic workflow; LOD, limit of detection; mNGS, metagenomic next-generation sequencing; RT-qPCR, reverse transcription quantitative polymerase chain reaction.

Table S2. Demographic information and clinical information of the clinical cohorts

| Characteristics | Value (proportion) | |
| --- | --- | --- |
|  | Retrospective cohort (n=64) | Prospective cohort (n=63) |
| Age, median years [range] | 59 [1-92] | 48 [6-68] |
| Male, No. (%) | 32 (50.00%) | 43 (68.25%) |
| Fever | 31 (48.44%) | 63 (100%) |
| Cough | 36 (56.25%) | 29 (46.03%) |
| Fatigue | 18 (28.13%) | 41 (65.08%) |
| Myalgia | 12 (18.75%) | 43 (68.25%) |
| Chills | 2 (3.13%) | 55 (87.30%) |
| Anorexia | 6 (9.38%) | 39 (61.90%) |
| Pharyngodynia | 2 (3.13%) | 52 (82.54%) |
| Nausea and vomiting | 2 (3.13%) | 6 (9.52%) |
| Diarrhea | 3 (4.69%) | 2 (3.17%) |
| Combined with chronic disease | 35 (54.69%) | 4 (6.35%) |
| Critical illness | 20 (31.25%) | 0 (0%) |
